# Recent Advances in Diversified Materials for Spinal Cord Injury Repair and Regeneration

**DOI:** 10.3390/gels12070566

**Published:** 2026-06-26

**Authors:** Yun Wang, Xingtao Wang, Yaqing Liu, Xueting Xuan, Yonghao Luo, Jihui Wang

**Affiliations:** 1 School of Life and Health Technology, Dongguan University of Technology, Dongguan 523808, China; yywang@dgut.edu.cn (Y.W.);; 2 School of Special Education and Rehabilitation Medicine, Shandong Medical and Pharmaceutical University, Yantai 264003, China; 3 School of Medicine, Xinjiang College of Science & Technology, Korla 841000, China; 4 College of Chemistry and Chemical Engineering, Shihezi University, Shihezi 832000, China

**Keywords:** spinal cord injury, gel-based biomaterials, nerve regeneration, bioactive materials, hydrogel

## Abstract

Spinal cord injury (SCI) is a devastating central nervous system disorder that causes irreversible loss of sensory and motor functions below the lesion, seriously compromising patients’ daily activities and quality of life. Due to the inherent shortcomings of existing therapeutic strategies and the rapid progress of material engineering, developing advanced functional materials has emerged as a promising approach for SCI treatment. This review comprehensively summarizes the applications of polymeric materials, inorganic materials, bioactive materials, and composite biomaterials for SCI treatment and regeneration, with a focus on their underlying mechanisms, therapeutic performance, and research trends. Cumulative evidence indicates that these materials possess versatile biological functions and great application potential in facilitating nerve regeneration and tissue reconstruction after SCI. In short, in-depth understanding of material-based therapeutic systems can offer innovative references for the optimization of SCI treatment regimens. Nevertheless, more preclinical and translational investigations are still indispensable to accelerate their clinical transformation and widespread practical use.

## 1. Introduction

### 1.1. Overview of Spinal Cord Injury

SCI is a severe, debilitating central nervous system disorder. It commonly leads to irreversible motor impairment, bladder dysfunction, neuropathic pain, and sphincter as well as autonomic nervous system disorders in regions below the injured segment [1,2,3]. The pathophysiologic process of SCI consists of two consecutive stages: primary mechanical damage and secondary inflammatory cascade reactions. The primary injury refers to direct and acute mechanical destruction of spinal cord tissue [4]. The second stage involves inflammatory responses, which triggers the activation of resident microglia and astrocytes, alongside the infiltration of peripheral macrophages and neutrophils. Such excessive inflammatory reactions further induce metabolic disturbance and functional deterioration of neurons and glial cells, ultimately exacerbating secondary tissue damage at and surrounding the lesion site [5,6,7].

The pathophysiology following SCI involves a series of complex and dynamic molecular mechanisms. The initial mechanical insult damages neurons, disrupts the spinal cord vascular system, and compromises the blood–spinal cord barrier (BSCB). Subsequent secondary injury processes further destroy spinal cord tissue, creating a microenvironment at the lesion site that is unfavorable for neuronal regeneration [8].

**Figure 1:**
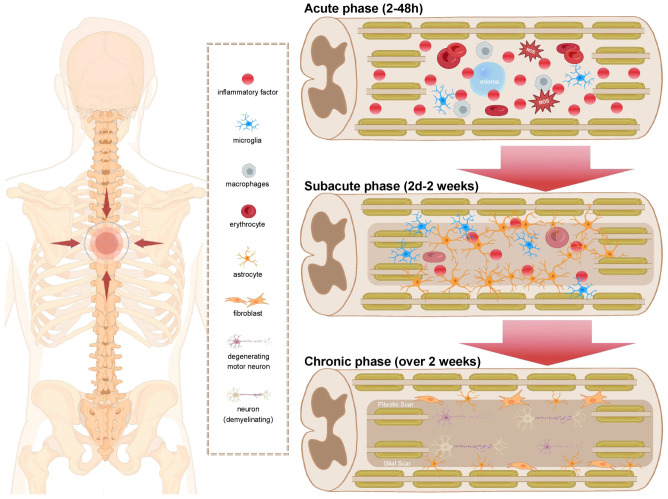
Schematic illustration of the temporal pathophysiological progression after SCI.

As depicted in Figure 1, the pathological evolution of SCI can be sequentially divided into acute, subacute, and chronic phases, each characterized by distinct cellular and molecular events. In the acute phase (2–48 h post-injury), multiple adverse events emerge, including excessive free radical accumulation, neurotransmitter-mediated excitotoxicity, calcium overload, ion imbalance, and lipid peroxidation [9]. Parenchymal hemorrhage upregulates pro-inflammatory factors and recruits abundant inflammatory cells, which exacerbate edema and tissue deterioration. Meanwhile, impaired vascular autoregulation induces persistent spinal cord ischemia, eventually resulting in massive neuronal loss [10]. During the subacute phase (2 days–2 weeks), continued ischemia in the spinal cord tissue triggers various specific mechanisms such as apoptosis, pyroptosis, demyelination, Wallerian degeneration, and axonal retraction, all of which inhibit neural repair after SCI [11]. Simultaneously, astrocytes proliferate, forming an enlarged and tightly packed network that acts as a physical barrier to nerve regeneration [8]. Activated astrocytes also secrete inhibitory molecules, such as chondroitin sulfate proteoglycans, which further impede spinal cord repair. As SCI progresses into the chronic phase (beyond 2 weeks post-injury), the formation of glial scars, increased extracellular matrix (ECM) deposition, and Nogo receptor-mediated inhibition of neuronal growth [12] make neural recovery and improvement of the microenvironment even more challenging.

### 1.2. Clinical Challenges Key Requirements for SCI Repair

The management of SCI mainly encompasses surgical intervention and pharmacotherapy. From a surgical perspective, decompressive surgery administered within 24 h plays a pivotal role. This therapeutic strategy aims to eliminate persistent compressive force on the spinal cord, boost neuroprotective effects following SCI, and reconstruct the stability of injured spinal segments. Nevertheless, such surgical procedures are invasive in nature, and there remains no unified consensus regarding the optimal surgical timing [13].

In pharmacological therapy, methylprednisolone serves as a core therapeutic agent. After SCI, systemic immune activation triggers massive release of inflammatory mediators, which further aggravates pathological damage to spinal cord tissues [9]. By inhibiting inflammatory cell aggregation, activation and mediator release, methylprednisolone relieves inflammatory injury. It also suppresses local lipid peroxidation and hydrolysis to prevent cell membrane disruption. Given its narrow 8 h therapeutic time window and potential risks of gastrointestinal bleeding and wound infection caused by high-dose intravenous use, the drug remains controversial in clinical practice with obvious application restrictions [14,15].

Thus, it is clear that relying solely on traditional surgical and pharmacological treatments is insufficient for effectively managing SCI. Given the complex and hostile local microenvironment following SCI, which impedes natural tissue regeneration, exploring alternative therapeutic strategies is essential. This highlights an opportunity for various biomaterials to play a role in spinal cord repair [16].

**Figure 2:**
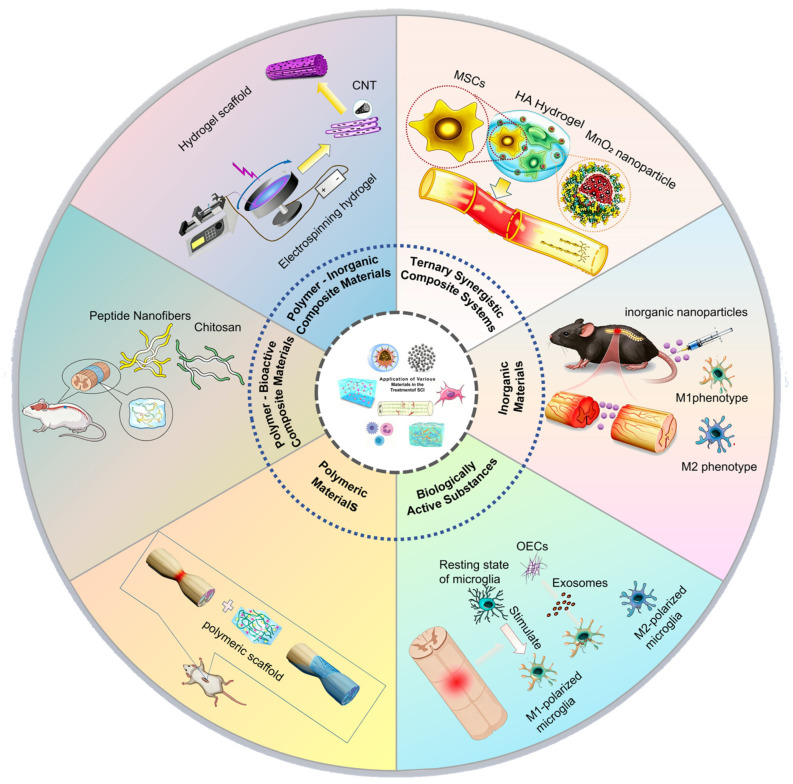
Application strategies of biomaterials for SCI repair.

While lesion-bypassing neural interfaces can effectively restore motor function post-SCI [17,18], the restoration of proprioception and voluntary movement relies heavily on further elucidation of cellular and molecular mechanisms underlying spinal cord repair. As shown in Figure 2, specialized biomaterials for spinal cord repair emerge as an innovative intervention. They can reconstruct a supportive microenvironment to span injury gaps, promote axonal regrowth and alleviate secondary injury, which greatly facilitates comprehensive neurological recovery.

## 2. Polymeric Materials

**Figure 3:**
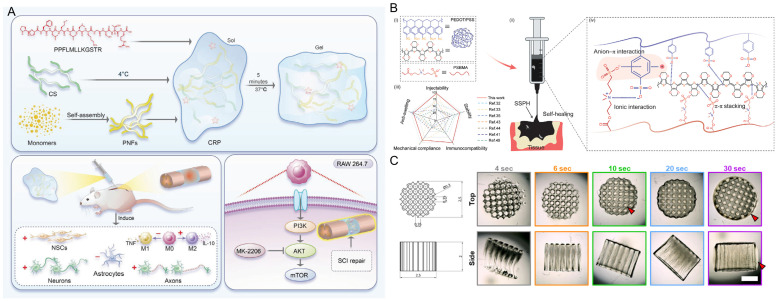
Representative polymeric biomaterials for SCI repair. (**A**) Thermosensitive chitosan/RADA_16_/neuropeptide hydrogels promote neural repair via PI3K/AKT/mTOR signaling [22]. (**B**) Conductive SSPH hydrogels provide stable bioelectronic interfaces for neural recording and stimulation [23]. (**C**) 3D-printed GelMA–PEGDA scaffolds guide axonal growth and promote functional recovery [24].

Polymeric materials can be derived from both natural polymers and synthetic polymers [19]. The major classifications along with their characteristics are summarized in Table 1. During their application (Figure 3), natural and synthetic polymeric materials each possess distinct advantages and limitations. In the treatment of SCI, polymeric materials often serve dual roles: on one hand, they act as scaffolds, providing essential physical support for nerve regeneration and promoting cell adhesion and proliferation; on the other hand, they function as drug carriers, enabling controlled release and targeted delivery of therapeutic agents [20,21].

Natural polymeric materials, such as certain ECM components, exhibit inherent bioactivity and a native ligand environment that allow for natural remodeling. As a result, they integrate well with host tissues after transplantation, facilitating the transition from experimental research to clinical applications [25,26]. In contrast, synthetic polymeric materials are characterized by low levels of impurities, pathogens, or contaminants, as well as reduced batch-to-batch variability. They demonstrate properties such as biodegradability, non-inflammatory behavior, and non-toxicity [27]. Moreover, the mechanical and physical properties of synthetic polymeric materials can be tailored through design [28].

Polymeric materials can also form three-dimensional network structures via chemical or physical crosslinking, thereby forming hydrogels capable of retaining large amounts of water molecules [29]. Hydrogels exhibit excellent biocompatibility, degradability, tunable physical structures, high porosity, and strong permeability, which are conducive to cell adhesion, migration, and growth. Their high water content and micro-/nanoporous structure make them ideal carriers for factors and cells, while also providing a site for nutrient metabolism and material exchange. Additionally, injectable hydrogels are suitable for contusion, compression, complete transection, and hemisection models, where they self-form into scaffold shapes that conform to the injury structure [30,31].

**Table 1:** Classification and characteristics of polymeric materials.

Category	Material/System	Advantages	Limitations	Main Roles in SCI Repair
Synthetic polymers	Polycaprolactone (PCL)	PCL has good mechanical strength, flexibility, biodegradability, low toxicity, and favorable biocompatibility.	PCL is relatively hydrophobic and has limited intrinsic bioactivity, which may restrict cell adhesion and neural integration without surface modification. Its slow degradation rate may also delay tissue remodeling.	Provides mechanically stable scaffolds, nerve conduits, and 3D-printed structures for axonal guidance and long-term structural support [32].
	Polylactic acid (PLA)	PLA is metabolizable and absorbable in vivo and exhibits good mechanical processability.	PLA is relatively brittle, and its acidic degradation products may aggravate local inflammation if degradation is not well controlled.	Used for electrospun fibers, porous scaffolds, and micro/nanostructured guidance systems to support axonal regeneration [33,34].
	Poly(lactic-co-glycolic acid) (PLGA)	PLGA has tunable degradation kinetics, good processability, and established use in controlled drug delivery systems [35].	Acidic degradation products and relatively limited cell-adhesive bioactivity may require combination with natural polymers or bioactive molecules.	Used as structural scaffolds, electrospun fibers, nerve guidance conduits, and composite matrices to provide mechanical support, guide axonal regeneration, reduce glial scar formation, and promote endogenous neural stem cell recruitment and functional recovery after SCI [36].
	Polyethylene glycol (PEG)/PEGDA	PEG-based polymers show good hydrophilicity, tunable crosslinking, and controllable mechanical properties.	PEG has limited intrinsic cell adhesion and biological signaling capacity unless modified with peptides or ECM-derived components.	Used to construct injectable, photocrosslinked, and 3D-printed hydrogels for lesion filling, cell encapsulation, and controlled release [37].
	Polyvinyl alcohol (PVA)	PVA is water-soluble, tunable, and highly biocompatible, making it suitable for hydrogel-based materials [38].	PVA has limited intrinsic bioactivity and weak cell-adhesive properties, requiring modification or combination with bioactive components.	Serves as a flexible hydrogel matrix for drug delivery, cell encapsulation, and sustained release in SCI repair.
Natural polymers	Collagen	Collagen is a major ECM component with inherent bioactivity, good biocompatibility, and cell-adhesive motifs that support cell attachment, migration, and tissue remodeling.	Collagen usually has insufficient mechanical strength and rapid degradation; animal-derived collagen may also show batch variability or immunogenic risk.	Acts as an ECM-mimicking scaffold to support cell infiltration, axonal growth, and neural tissue reconstruction [39].
	Hyaluronic acid (HA)	HA exhibits molecular-weight-dependent biological effects. High-molecular-weight HA promotes nerve regeneration, structural repair, and anti-inflammatory responses through its ECM-mimicking properties [40,41].	HA has weak mechanical strength and rapid degradation. Low-molecular-weight HA fragments may activate inflammatory signaling if molecular weight, dose, and delivery mode are not carefully controlled.	Serves as an injectable hydrogel matrix for cell delivery, exosome retention, angiogenesis regulation, and neural repair [42].
	Chitosan	Chitosan has structural similarity to glycosaminoglycans and exhibits good biocompatibility, biodegradability, antibacterial activity, and chemical modifiability [43,44].	Chitosan has limited solubility under physiological pH and relatively weak mechanical strength unless chemically modified or combined with other polymers.	Used to construct injectable, thermosensitive, and adhesive hydrogels for drug delivery, immune modulation, axonal regeneration, and infection prevention.
	Alginate	Alginate exhibits good biocompatibility, biodegradability, non-antigenicity, and chelating properties [45].	Alginate has limited intrinsic cell-adhesive ability and relatively weak mechanical stability, often requiring peptide modification or combination with other polymers.	Serves as a mild gelation matrix for cell encapsulation, controlled release, and composite scaffold construction for neural repair [46].
	Silk fibroin (SF)	Silk fibroin has good biocompatibility, tunable mechanical strength, biodegradability, and processability into fibers, films, and hydrogels.	Its degradation behavior and stiffness need to be carefully controlled to match soft spinal cord tissue.	Used for mechanically stable scaffolds, aligned fibrous structures, and injectable hydrogels to guide axonal regeneration and tissue remodeling [47].
	Gelatin/GelMA	Gelatin and GelMA retain bioactive motifs derived from collagen and show good biocompatibility, photocrosslinkability, and tunable mechanical properties.	Gelatin-based systems may have weak mechanical stability and fast degradation without sufficient crosslinking; photocrosslinking conditions should be optimized to avoid cell damage.	Used for injectable, photocrosslinked, and 3D-printed scaffolds for cell encapsulation, axonal guidance, and controlled release [48].
Composite polymers	GelMA + PEGDA	Combines the excellent cell-adhesive bioactivity of GelMA with the tunable mechanical strength, structural stability, and photocrosslinking properties of PEGDA, enabling the fabrication of robust and biomimetic scaffolds for neural regeneration [49].	GelMA/PEGDA hydrogels suffer from crosslinking-dependent imbalance: excessive crosslinking increases stiffness and reduces cell adhesion and neurite growth, while also limiting viability and diffusion; insufficient crosslinking weakens structural stability, compromising scaffold function in SCI repair.	GelMA/PEGDA hydrogels primarily serve as photocrosslinkable and mechanically tunable scaffold platforms for structural reconstruction, lesion cavity filling, and 3D bioprinting applications [50].
	HA + PEG	Combines the ECM-mimicking and anti-inflammatory properties of HA with the controllable crosslinking density and mechanical stability of PEG hydrogels, creating a supportive microenvironment for neural survival and regeneration [37].	The HA/PEG composite system exhibits relatively limited intrinsic biofunctionality, with particularly insufficient performance in immunomodulation, neuroprotection, and dynamic microenvironment regulation. Consequently, it is unable to actively intervene in the complex pathological cascades following spinal cord injury, including inflammatory responses, oxidative stress, and glial scar formation.	HA/PEG hydrogels mainly act as ECM-mimicking, injectable scaffold platforms that provide structural support, hydrated microenvironments, and localized delivery of cells and bioactive factors [51].

## 3. Inorganic Materials

Numerous types of inorganic materials exist, yet those for SCI treatment need to satisfy distinct requirements (Table 2). They need superior biocompatibility with low immunogenicity and good cell affinity, as well as suitable mechanical parameters including strength, toughness and elastic modulus. For injectable and in situ forming hydrogels used in SCI repair, critical design parameters include gelation windows of 5–30 min at physiological temperature, equilibrium swelling ratios of 5–15-fold (to avoid excessive post-injection compression on delicate spinal cord tissue), and degradation kinetics tuned to 2–12 weeks to match the timeline of neural tissue repair. These parameters are carefully balanced against the native spinal cord elastic modulus (0.1–1.0 kPa) to ensure mechanical compatibility and minimize secondary injury. They should also exert favorable bioactivity to boost nerve regeneration and angiogenesis. Controllable biodegradability matching tissue repair progress is also essential, alongside functional physical properties like conductivity and electromagnetic and optical properties. Adequate drug loading and sustained release capacity help boost curative effects and lower side effects. In SCI intervention, inorganic materials can reinforce composite systems to optimize mechanical, electrical and magnetic performances and offer structural support for neural restoration. They can also act as drug carriers, enabling precise drug release and thereby improving therapeutic outcomes [52,53,54]. Redox-active nanomaterials, including manganese dioxide (MnO_2_), cerium oxide (CeO_2_), and selenium-based nanoparticles, play particularly important roles in regulating the oxidative microenvironment after SCI. MnO_2_ can consume excessive ROS while simultaneously generating oxygen, thereby alleviating oxidative stress and local hypoxia. CeO_2_ nanoparticles function as nanozymes through reversible Ce^3+^/Ce^4+^ redox cycling, providing long-lasting antioxidant activity and protecting neural cells from oxidative damage. Similarly, selenium nanoparticles exert potent antioxidative and anti-inflammatory effects through the regulation of endogenous antioxidant pathways, helping to suppress secondary injury cascades.

**Figure 4:**
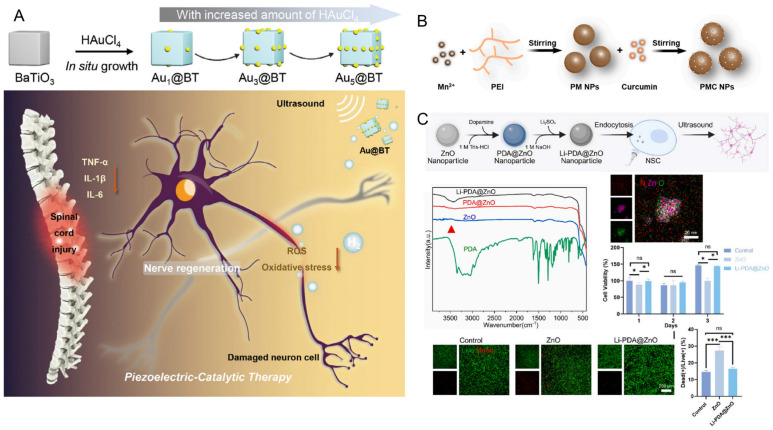
Representative inorganic nanomaterials for SCI repair. (**A**) Au@BT nanoparticles generate hydrogen via ultrasound-driven piezocatalysis for acute SCI treatment [55]. (**B**) PMC nanoparticles act as ROS-scavenging and anti-inflammatory nanozymes [56]. (**C**) Li-PDA@ZnO nanoparticles promote neural stem cell differentiation under ultrasound stimulation, they possess excellent biocompatibility with no significant difference between the control and Li-PDA@ZnO groups. Statistical significance: * indicates *p* < 0.05 and *** indicates *p* < 0.001 compared with the control group [57].

As illustrated in Figure 4, metal-based inorganic nanoparticles stand out among diverse inorganic materials and exhibit remarkable application potential in SCI repair. For instance, ultrasound-responsive gold-decorated barium titanate (Au@BT) piezoelectric nanoparticles can effectively inhibit oxidative stress and neuronal apoptosis [55]. PEI-Mn@curcumin (PMC) nanoparticles are engineered as multifunctional nanozymes that exert ROS scavenging and anti-inflammatory effects to achieve neuroprotection [56]. Moreover, green-synthesized zinc nanoparticles are capable of protecting motor neurons and alleviating glial scar formation [48]. Collectively, these metallic inorganic nanomaterials integrate antioxidant, anti-inflammatory, neuroprotective and neural circuit-modulating properties, thus emerging as a highly promising combinatorial strategy for SCI repair.

**Table 2:** Classification and characteristics of inorganic materials.

Classification	Characteristics
Gold nanoparticles	Gold nanoparticles (AuNPs) have good biocompatibility and stability and can be used as drug delivery carriers. AuNPs can also achieve targeted therapy through surface modification, improving the therapeutic effect of drugs [58,59]. Additionally, AuNPs can generate heat under near-infrared light irradiation for photothermal therapy of SCI [60].
Silver nanoparticles	Silver nanoparticles (AgNPs) exhibit strong broad-spectrum antibacterial activity and can help prevent infections after SCI. When incorporated into biomaterial matrices at controlled low concentrations, AgNPs provide localized antimicrobial effects and mild signaling cues that support neurite outgrowth. Importantly, free Ag^+^ ions released from uncoated nanoparticles can be cytotoxic at elevated doses; therefore, AgNPs are typically embedded within protective polymer networks [61,62].
Carbon nanotubes	Carbon nanotubes (CNTs) have excellent mechanical properties and electrical conductivity and can be used as nerve scaffold materials. CNTs can also carry drugs and growth factors to promote nerve regeneration. In addition, CNTs can generate heat through near-infrared light excitation for photothermal therapy of SCI [63,64].
Graphene	Graphene sheets possess ultrahigh tensile strength (~130 GPa), Young’s modulus (~1 TPa), and exceptional electrical conductivity (~10^6^ S m^−1^). When incorporated into scaffolds at low concentrations (0.1–1 wt%), graphene significantly enhances mechanical reinforcement, imparts electrical conductivity for external stimulation therapies, and promotes axonal outgrowth and neural signal transmission. Its high surface area also facilitates drug/growth factor loading and photothermal therapy under near-infrared irradiation [63,65].
Silicon dioxide	Silicon dioxide (SiO_2_) is antibacterial, anti-inflammatory, and promotes healing, can be used as a drug carrier, and has certain biocompatibility [66].
Manganese dioxide	MnO_2_ has been introduced into hydrogel systems to regulate the ROS-rich microenvironment after SCI. MnO_2_ nanoparticle-dotted hydrogels can consume excessive ROS and alleviate local hypoxia through their oxygen-generating activity, thereby creating a more favorable microenvironment for mesenchymal stem cell-mediated spinal cord repair [67].
Molybdenum disulfide	Molybdenum disulfide (MoS_2_) is usually used as a functional conductive component rather than a simple filler. In MoS_2_/graphene oxide/polyvinyl alcohol nanocomposite hydrogels, MoS_2_ contributes to electrical conductivity and photothermal responsiveness, helping the hydrogel provide a more permissive interface for neural repair after SCI [68].
Iron oxide nanoparticles	Iron oxide nanoparticles are mainly used for magnetic regulation and targeted delivery in SCI repair. In magnetic nanoparticle- and methylprednisolone-based neural stem cell delivery systems, the magnetic component improves cell localization and retention at the lesion site, while methylprednisolone provides anti-inflammatory protection for the transplanted cells [69].
Cerium oxide	CeO_2_ functions as a redox-active nanozyme through reversible Ce^3+^/Ce^4+^ cycling. In nitric oxide-releasing mesoporous hollow CeO_2_ nanozyme-based hydrogels, CeO_2_ is used to scavenge ROS and regulate oxidative stress, while nitric oxide release and neural stem cell loading jointly support spinal cord tissue repair [70].
Zinc oxide	Zinc oxide (ZnO) is valued for its piezoelectric and ultrasound-responsive properties. In Li-PDA@ZnO nanoparticle-containing biohydrogels, ultrasound stimulation activates ZnO-based electromechanical cues, which promote neural stem cell differentiation and enhance neural regeneration after SCI [57].
Selenium-based nanomaterials	Selenium-based nanomaterials are mainly applied for antioxidative and anti-inflammatory regulation. Selenium nanoparticles derived from Proteus mirabilis YC801 were reported to reduce oxidative stress and inflammatory responses after SCI, thereby protecting neural tissue and promoting nerve repair [71].
Hydroxyapatite	Hydroxyapatite is not only a structural reinforcing component but can also serve as a bioactive platform for cell-based repair. Multifunctional hydroxyapatite nanobelt “haystacks” integrated with neural stem cell spheroids provide a supportive microenvironment for cell survival, neural differentiation, and rapid spinal cord repair [72].

## 4. Biologically Active Substances

**Figure 5:**
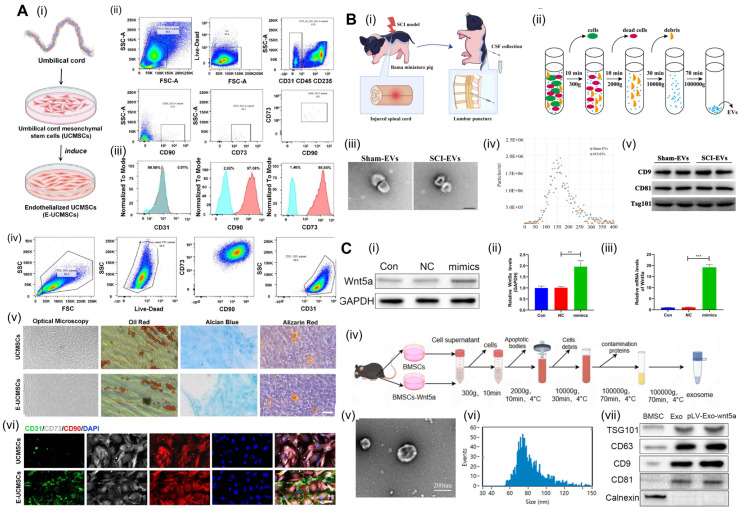
Representative biologically active substances for SCI repair. (**A**) RGD-modified UCMSC exosomes promote angiogenesis and BSCB stabilization [83]. (**B**) SCI-induced cerebrospinal fluid extracellular vesicles enhance vascular regeneration via PI3K/AKT signaling [84]. (**C**) Wnt5a-engineered BMSC exosomes regulate inflammation and neural differentiation via NF-κB signaling [85]. Statistical significance: ** indicates *p* < 0.01 and *** indicates *p* < 0.001 compared with the control group.

In the field of biomedicine, a variety of biologically active substances have demonstrated critical roles in the treatment of SCI (Table 3). These substances primarily include cells, exosomes, and growth factors. Neural stem cells (NSCs) and mesenchymal stem cells (MSCs), as seed cells with significant regenerative potential, exhibit self-renewal capacity and multi-lineage differentiation potential. These cells can differentiate into neurons, oligodendrocytes, and astrocytes, thereby promoting neural regeneration and repair by replenishing lost cellular components in the injured spinal cord [73,74]. Supporting cells such as olfactory ensheathing cells (OECs) provide structural and trophic support for regenerating axons. OECs facilitate endogenous remyelination by optimizing the local microenvironment, protecting myelinating glia (oligodendrocytes and Schwann cells), and secreting various growth factors and ECM components, thereby enhancing nerve conduction velocity without directly forming the myelin sheath [75]. Exosomes, nanoscale vesicles secreted by cells, contain abundant bioactive molecules, including proteins, nucleic acids, and lipids [76]. In SCI therapy, exosomes play a pivotal role in signal transduction and intercellular communication [77]. Compared to cell transplantation, exosome-based therapies offer several advantages, including low immunogenicity, ease of storage and transportation, and absence of tumorigenic risks [78]. As a promising cell-free therapeutic approach, exosomes circumvent potential issues such as immune rejection and ethical concerns associated with cell transplantation. They can be delivered precisely to the injured spinal cord site via intravenous or local injection, exerting therapeutic effects [79]. Growth factors, including nerve growth factor (NGF), brain-derived neurotrophic factor (BDNF), and glial cell line-derived neurotrophic factor (GDNF), are essential in SCI treatment [80]. These factors stimulate the growth, survival, and differentiation of neural cells, promote axonal elongation, and enhance neuronal function, thereby laying the foundation for neural regeneration [80,81,82]. Moreover, growth factors modulate inflammatory responses and angiogenesis. On one hand, they suppress the activation of inflammatory cells and the release of pro-inflammatory cytokines, reducing inflammation and stabilizing the microenvironment for neural repair. On the other hand, they promote the proliferation and migration of vascular endothelial cells, improving blood supply to the injured area and providing essential nutrients and oxygen for neuronal survival and regeneration [80]. As depicted in Figure 5, exosome-based therapies have emerged as promising cell-free strategies for SCI repair. Arg-Gly-Asp (RGD)-modified exosomes derived from endothelialized umbilical cord mesenchymal stem cells (UCMSCs) specifically target neovascular endothelial cells, enhance angiogenesis, stabilize the BSCB, and effectively promote neurological recovery [83]. Additionally, cerebrospinal fluid extracellular vesicles induced by SCI can significantly boost vascular regeneration and functional recovery by activating the PI3K/AKT signaling pathway [84]. Meanwhile, Wnt5a-engineered bone marrow mesenchymal stem cell (BMSC) exosomes exert anti-inflammatory and neuroregenerative effects by inhibiting the NF-κB pathway, driving microglia toward the M2 phenotype, facilitating neuronal differentiation, and reducing astrogliosis [85].

## 5. Composite Materials

Polymeric materials, with their excellent biocompatibility and degradability, are considered ideal candidates for long-term therapeutic applications [98]. Fine-tuning their molecular structures enables effective control over the mechanical properties and degradation rates of these materials [99]. In the fields of nerve repair and drug delivery systems, polymeric materials exhibit outstanding performance, particularly in applications requiring high flexibility and biocompatibility. However, pure polymeric materials often exhibit insufficient mechanical properties, poor electrical conductivity, and limited therapeutic efficacy. They serve as supporting matrices to provide appropriate physical microenvironments for cells at the implantation sites [99,100,101].

Inorganic materials such as CNTs and graphene are known for their outstanding mechanical strength, electrical conductivity, and bioactivity. They provide the necessary rigidity and structural stability for composite materials and demonstrate exceptional performance in the field of electrical stimulation-based repair. Raw inorganic nanoparticles may exhibit dose-dependent cytotoxicity or chronic tissue accumulation risks due to ion release and limited biodegradability. These limitations are precisely the reason why inorganic nanomaterials for SCI applications must be surface-modified, green synthesized, or embedded at low concentrations within protective polymer matrices to achieve the superior biocompatibility and controllable degradation profiles required for safe and effective neural repair [102,103].

**Figure 6:**
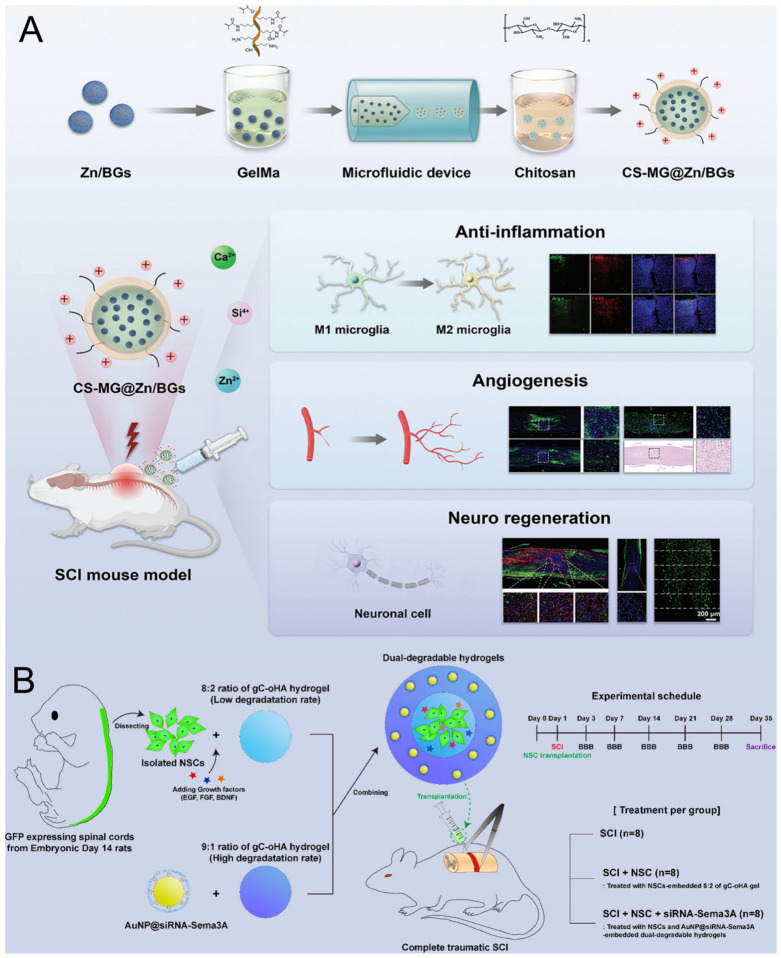
Representative composite biomaterials for SCI repair. (**A**) Chitosan-modified hydrogel microspheres promote immunomodulation, angiogenesis, and axonal regeneration [106]. (**B**) Dual-degradable hydrogels co-delivering Sema3A siRNA-loaded AuNPs and neural stem cells enhance synaptic integration and locomotor recovery after SCI [107].

Biologically active substances, with their unique physiological activity and targeting ability, exhibit significant promise in the treatment of SCI. Through specific mechanisms of action, biologically active substances can promote nerve regeneration, inhibit inflammatory responses, and modulate cellular microenvironments. However, the harsh microenvironment of SCI may reduce the activity of biologically active substances. Additionally, their poor stability and short half-life in vivo limit the persistence and efficacy of their therapeutic effects [104,105]. As shown in Figure 6, diverse combinations of the three aforementioned materials enable functional complementarity and thus produce synergistic effects in SCI therapy.

### 5.1. Polymer-Inorganic Composite Materials

When fabricating composites via blending polymers with inorganic substances, polymers generally act as scaffold matrices, while inorganic components exert indispensable functions within such composite systems. Primarily, inorganic fillers can reinforce the mechanical strength and electrical properties of polymer substrates, as well as endow them with magnetic responsiveness, further stabilizing the structural framework for neural repair.

Collagen, the most prevalent protein in the human body, is mainly distributed in the skin, bone, tendon and cartilage tissues. In SCI therapy, collagen is widely adopted to fabricate tissue-engineered scaffolds and serves as a favorable polymeric matrix to facilitate neural tissue repair and regeneration [95,108]. Nevertheless, collagen suffers from inferior mechanical strength, which renders it unable to withstand compression and tension at spinal cord injury sites. Such limitation easily causes scaffold deformation or collapse, ultimately disrupting the microenvironment for neural regeneration [108].

In contrast, graphene possesses ultrahigh tensile strength and stiffness, together with superior electrical and thermal conductivity [109]. Accordingly, Gopal Agarwala’s team rationally integrated the merits of the two materials. They uniformly dispersed amine-functionalized graphene in aqueous solution and successfully synthesized graphene-crosslinked collagen cryogels with varied graphene concentrations. Experimental results confirmed that graphene incorporation markedly boosted the mechanical performance of collagen cryogels, achieving outstanding compression resistance and structural stability. Meanwhile, graphene imparted electrical conductivity to the cryogels, enabling therapeutic improvement via external electrical stimulation [110]. The incorporation of graphene or CNTs imparts electrical conductivity to the cryogels, enabling external electrical stimulation. At the molecular level, these conductive cues are transduced via activation of voltage-gated calcium channels (VGCCs) on neuronal and stem cell membranes, leading to intracellular Ca^2+^ influx. This triggers downstream signaling cascades, including CREB phosphorylation and upregulation of neurotrophic factors such as BDNF and NGF. Consequently, conductive scaffolds promote neuronal differentiation, axonal outgrowth, and synaptic formation more effectively than non-conductive controls, as demonstrated in graphene–collagen cryogel studies. Relevant evidence has verified that electrical stimulation can facilitate cell proliferation, differentiation and migration, restore neural signal conduction, and accelerate nerve regeneration [111,112], which is consistent with the conclusions drawn by Agarwala et al.

The spinal cord presents a highly ordered anatomical structure, where axons and myelin sheaths are arranged longitudinally. A critical design consideration for aligned guidance hydrogels is the prevention of anisotropic swelling that could collapse oriented microchannels upon hydration in vivo. Researchers address this challenge by employing covalent crosslinking strategies (e.g., in GelMA or silk fibroin matrices), optimizing equilibrium swelling ratios to <8-fold, and utilizing photo-crosslinked or thermosensitive polymers with rapid gelation kinetics. These approaches preserve channel integrity and maintain directional guidance cues for regenerating axons while allowing necessary nutrient diffusion [113,114,115,116]. Multichannel bridging architectures have been proven to effectively facilitate directional axonal outgrowth [117]. On this basis, Courtney M. Dumont and colleagues took advantage of inherent axonal growth characteristics and fabricated polymeric scaffold conduits and oriented bridge structures with linear channels by mixing microspheres with photoinitiators. These constructs possess favorable cell infiltration and tissue integration capacities within a hyaluronic acid (HA) matrix, and their internal oriented channels can precisely guide ordered axonal regeneration [113].

Recent advances have revealed that aligned fibrous architectures better mimic the extracellular matrix structure of native neural tissues, and such oriented topological features are essential for directing axonal regeneration and synaptic reformation [114]. Accordingly, Shenglian Yao et al. fabricated carbon nanotube-incorporated aligned fibrous composite scaffolds via rotating liquid bath electrospinning. This design realized the structural evolution from linear channels to biomimetically aligned fibers and simultaneously conferred electrical conductivity upon polymeric scaffolds.

When integrated with electrical stimulation, this strategy facilitates the proliferation and adhesion of PC12 cells and further synergistically boosts the differentiation of neural stem cells (NSCs) [118]. Likewise, Chun-Yi Yang and colleagues fabricated aligned fibrous composite Nrf2 antioxidant pathway polymer scaffolds embedded with Fe_3_O_4_ magnetic nanoparticles (MNPs) via electrospinning, which imparted magnetic field (MF) responsiveness to the scaffolds. In combination with magnetic field stimulation, such scaffolds could accelerate PC12 cell proliferation and nerve growth factor (NGF) secretion, thus achieving synergistic promotion of neural tissue regeneration [115].

In another study, Arianna Rossi constructed magnetic collagen bundles (MCollB) by conjugating type I collagen with MNPs. By applying remote low-intensity magnetic stimulation, the orientation of MCollB within hydrogel matrices could be precisely regulated, enabling the fabrication of aligned structured hydrogels [116].

Apart from the hybridization of inorganic and polymeric biomaterials, several studies have focused on macrophage modulation for SCI therapy. Xiaoliang Cui et al. designed an injectable ZnMn@silk fibroin (SF) hydrogel for SCI repair by incorporating ZnMn layered double hydroxides (LDHs) into a silk fibroin matrix. ZnMn-LDHs are capable of neutralizing local acidic microenvironments and achieving a sustained release of Zn^2+^ and Mn^2+^, which strengthens metal ion-mediated regulation of macrophage polarization. Mechanistically, this system elevates the expression levels of SOD1 and SOD2 in injured lesions, upregulates metallothionein (MT) expression via activating the MTF-1 pathway, triggers the Nrf2 antioxidant pathway and suppresses the NF-κB inflammatory signaling pathway. Collectively, it relieves the early inflammatory response after SCI and optimizes the local immune microenvironment [119].

In terms of the therapeutic function of Mn^2+^, Yuyu Sun’s team developed a biomineralized MnO_2_ nanoparticle-based thermosensitive hydrogel composed of chitosan, β-glycerophosphate (β-GP), polyvinyl alcohol (PVA) and glutaraldehyde (GA). The incorporated MnO_2_ nanoparticles can catalytically decompose hydrogen peroxide to generate oxygen, ameliorating the hypoxic microenvironment of damaged spinal cord tissues. Moreover, they restrain oxidative stress-induced neuronal ferroptosis through regulating the SIRT1 signaling pathway, thereby diminishing neuronal apoptosis and facilitating neural functional repair after SCI [120].

Leveraging the thermosensitive property and acidic microenvironment targeting capability, Ruofei Wang et al. exploited the characteristic that iron sulfide (greigite Fe_3_S_4_) releases hydrogen sulfide (H_2_S) under acidic conditions and synthesized ferrofluid hydrogels (FFH) by reacting Fe_3_S_4_ colloids with carboxymethyl chitosan and AuNPs. Fe_3_S_4_ particles possess magnetic field-induced directional alignment ability, endowing the hydrogel with excellent anisotropic structure. Under acidic pathological conditions, Fe_3_S_4_ enables slow and sustained H_2_S release in vivo, avoiding adverse effects caused by abrupt excessive H_2_S release and poor drug stability. The steadily released H_2_S alleviates local oxidative stress, reduces lipid peroxidation accumulation, and exerts protective effects on neurons and nerve fibers at SCI lesions [121].

Additionally, black phosphorus quantum dots have also been explored for SCI treatment. Xie et al. constructed polymer scaffolds co-loaded with black phosphorus quantum dots and gallic acid. In vivo animal experiments verified that the composite scaffolds could inhibit post-traumatic neuronal apoptosis and accelerate neuronal regeneration by reactivating cell cycle progression and modulating the AKT signaling cascade [122].

Overall, the combination of polymeric and inorganic biomaterials can effectively optimize the inherent physicochemical properties of polymer matrices and endow them with conductivity, magnetic responsiveness and other novel functions, ultimately realizing synergistic therapeutic effects for spinal cord injury repair.

### 5.2. Polymer-Bioactive Composite Materials

When polymeric materials are combined with biologically active substances to form composite materials, the polymeric materials often serve as scaffolds and carriers, while the biologically active substances typically act as functional active centers. Polymeric material scaffolds generally employ two strategies to deliver biologically active substances to the damaged area. One strategy involves the in situ injection of a mixture of polymeric scaffold precursors and biologically active substances, which rapidly solidifies and is implanted at the SCI site to fill the voids in the injured spinal cord region, bridging the damaged area. During the self-degradation process of the scaffold, it provides contact guidance for the regeneration of neurons and axons. In this scenario, the polymeric material scaffold acts as a carrier for the biologically active substances, enabling the local enrichment and sustained release of seed cells. This ensures that the loaded cells can be delivered to the SCI environment in a timely manner, providing an appropriate microenvironment for the reconstruction of the nervous system [123]. The core advantage of this method lies in the close correlation between the mechanical properties and degradation rate of the polymeric material scaffold and the regeneration rates of neurons and axons. The other strategy involves implanting biologically active substances into a pre-designed scaffold, which is then transplanted to the SCI area. Such scaffolds usually have specific internal structures and geometries, with precisely controlled pore sizes, porosities, and mechanical strengths. However, the limitation of this strategy is that the adhesion between cells and the scaffold is difficult to control accurately, resulting in unsatisfactory sustained-release effects [124]. The adhesion between transplanted cells and scaffolds can be significantly stabilized through well-established surface functionalization strategies, including covalent grafting of RGD peptides, plasma or UV-ozone surface activation to introduce hydrophilic functional groups, and carbodiimide (EDC/NHS) chemical crosslinking. These approaches have been shown to increase cell adhesion strength by 2–4 fold, improve long-term engraftment, and enhance sustained-release efficacy of therapeutic cells in SCI models [125,126].

#### 5.2.1. Cell as Biologically Active Substance

At the current stage, a wide variety of cells have demonstrated great potential in neural repair treatment, including NSCs, MSCs, and embryonic stem cells (ESCs). Transplanted stem cells can differentiate into neurons and glial cells while secreting various cytokines to inhibit inflammatory responses and prevent cell apoptosis. Furthermore, they promote axonal regeneration and restore signal transmission between neurons, thereby improving functional recovery after SCI [127]. Specific matrix biophysical parameters are deliberately engineered to control NSC fate. Low elastic modulus hydrogels (<1 kPa) and aligned microchannel architectures (5–20 µm width) reduce mechanical cues that favor astrocytic differentiation (GFAP upregulation) while promoting neuronal lineage commitment. Conductive scaffolds (e.g., CNT- or graphene-containing) further activate voltage-gated calcium channels and downstream CREB/BDNF signaling pathways, steering NSCs toward functional neurogenesis and suppressing glial scar formation. These design principles are now explicitly summarized to guide future material engineering for targeted differentiation control. Yuan et al. developed a cell-adaptive dynamic covalent hydrogel reinforced with stem cells as a delivery vehicle for adipose-derived stem cells (ADSCs) to promote neuronal regeneration after SCI [125]. The dynamic network of the neurogenic hydrogel loaded with ADSCs provided an infiltration matrix for cells, promoting axonal growth in SCI rats. This approach enhanced motor-evoked potentials, hindlimb strength, and coordination after complete spinal cord transection. Fan et al. [127] combined gelatin with NSCs and observed that scaffolds with a low elastic modulus exhibited more robust neurite growth and pronounced neuronal differentiation. Moreover, transplantation of Gel/NSCs scaffolds reduced macrophage activation and inflammation levels in the injured area, decreasing cavity size. However, excessive deposition of collagen fibers produced by astrocytes led to glial scar formation, limiting neural plasticity and hindering endogenous tissue repair. Liu et al. [125] encapsulated NSCs in a three-dimensional chondroitin sulfate methacrylate scaffold. This method inhibited astrocytic differentiation of NSCs both in vitro and in vivo, reducing the formation of glial fibrillary acidic protein (GFAP) in the injured area. Similarly, Huang et al. confirmed that BMSCs suppressed GFAP generation in the injured region [128].

One of the major obstacles to cell transplantation is the excessive accumulation of ROS at the injury site, which compromises cell survival and therapeutic efficacy. To address this issue, Li et al. encapsulated BMSCs within a thioacetal-containing ROS-responsive polymeric scaffold. The scaffold effectively scavenged ROS, reduced oxidative stress and inflammatory cytokine expression, and improved the survival of transplanted BMSCs. In turn, the surviving BMSCs exerted potent paracrine effects through the secretion of neurotrophic and anti-inflammatory factors, further enhancing neuroprotection and tissue repair [129].

Similarly, Ying et al. developed a ROS-scavenging hydrogel based on TPA and laponite combined with dental pulp stem cells (DPSCs). While the hydrogel protected DPSCs from oxidative damage by regulating iron metabolism and inhibiting ferroptosis, DPSCs contributed to tissue regeneration through their antioxidative, angiogenic, and neurotrophic activities. This reciprocal interaction significantly enhanced the regenerative efficacy of the composite system [130].

In addition to improving the biochemical microenvironment, biomaterial scaffolds can provide structural support and guidance for neural regeneration. A composite patch employs a core–shell nanofiber architecture with differential degradation barriers: the outer layer degrades faster to release methylprednisolone in the acute phase, followed by sustained exosome release from the inner hydrogel core over 7–14 days. This temporally controlled delivery is governed by the layered structure and distinct degradation kinetics of the two components rather than simultaneous burst release [131].

Likewise, Xiao et al. engineered a DOPA-grafted chitosan-based self-healing hydrogel (CD/HRI), which provided a mechanically adaptive and immunomodulatory microenvironment for transplanted cells. The encapsulated cells further contributed to tissue repair through trophic support, thereby promoting axonal regeneration, synapse formation, and remyelination [132].

Building upon this strategy, Liu et al. incorporated neural stem cells (NSCs) and donepezil into a conductive self-healing hydrogel. The hydrogel facilitated NSC survival and differentiation by providing suitable porosity, conductivity, and controlled degradation, whereas NSCs contributed to neural regeneration through neuronal differentiation and trophic support [133].

Immune rejection remains another major challenge for stem cell transplantation. To overcome this limitation, Tian et al. developed an immunomodulatory hydrogel composed of aldehyde-modified oligochitosan, carboxymethyl chitosan, copper-doped carbon dots, and tacrolimus. The hydrogel enabled sustained local release of tacrolimus and provided mechanical protection for induced pluripotent stem cells (iPSCs), thereby improving cell survival and integration. In the self-healing and injectable hydrogel network, tacrolimus achieves local sustained release to exert immunosuppressive effects and prevent immune rejection by inhibiting T-cell activation [134]. Simultaneously, iPSCs served as a renewable source of pluripotent cells capable of differentiating into neural lineage cells and contributing to tissue reconstruction. Critically, the surrounding hydrogel matrix simultaneously provides mechanical protection, appropriate pore size for nutrient diffusion, and a supportive microenvironment that enhances iPSC survival, integration, and differentiation within the hostile SCI lesion site. Thus, the hydrogel and iPSCs functioned cooperatively to overcome immune barriers and enhance regenerative outcomes.

Furthermore, strategies combining cell transplantation with vascular regeneration have demonstrated considerable therapeutic potential. Guo et al. incorporated human DPSCs into pre-vascularized scaffolds and observed substantial improvements in angiogenesis, vascular perfusion, axonal regeneration, remyelination, and sensory recovery. The pre-vascularized scaffold improved oxygen and nutrient supply to the transplanted DPSCs, whereas DPSCs promoted angiogenesis and neuroregeneration through the secretion of pro-angiogenic and neurotrophic factors. The mutually reinforcing relationship between vascular reconstruction and cell-mediated repair significantly improved functional recovery [135].

Decellularized extracellular matrix (dECM)-based biomaterials have also attracted increasing attention because they retain native biochemical cues and closely mimic the neural microenvironment. Agarwal et al. developed an injectable hydrogel derived from decellularized porcine peripheral nerves as a carrier for Schwann cells. The dECM hydrogel provided an ECM-like niche that protected Schwann cells from inflammatory injury and supported their survival and proliferation. In return, Schwann cells promoted axonal guidance, remyelination, and neural regeneration, thereby maximizing the therapeutic benefits of the dECM-based system [136,137].

Collectively, these studies demonstrate that biomaterials and transplanted cells play complementary and interdependent roles in SCI repair. Biomaterial scaffolds and hydrogels provide structural support, protection from oxidative stress and inflammation, immunomodulation, and guidance for tissue regeneration, whereas transplanted cells actively participate in neural repair through differentiation, trophic factor secretion, angiogenesis, remyelination, and immune regulation. Therefore, the therapeutic efficacy of these systems arises not from biomaterials or cells alone, but from the synergistic interactions between them.

Despite these encouraging advances, several challenges remain. The complex pathological microenvironment following SCI continues to limit the long-term survival, integration, and therapeutic efficacy of transplanted cells. Moreover, the optimal cell source, transplantation strategy, and regulation of cell differentiation have not yet been fully established. Future studies should focus on enhancing cell survival, precisely regulating cell fate, optimizing biomaterial–cell interactions, and promoting long-term functional integration within the injured spinal cord to maximize functional recovery after SCI.

#### 5.2.2. Exosome as Biologically Active Substance

In recent years, with continuous advances in tissue engineering, tissue-engineered nerve grafts have been considered potential alternatives to autologous nerve grafts. The combination of polymeric scaffolds with encapsulated exosomes has attracted increasing attention for neural tissue repair. The three-dimensional network structure of hydrogels provides a stable release platform for exosomes, enabling sustained delivery of their bioactive cargos. This strategy can improve neurological dysfunction, reduce inflammatory responses, and enhance neuronal regeneration [138,139].

Adipose-derived mesenchymal stem cells (ADSCs) and their derived exosomes have shown considerable potential in SCI treatment. Zhou et al. demonstrated that ADSC-derived exosomes encapsulated in Pluronic F-127 hydrogel could be effectively protected and delivered. Pluronic F-127, composed of polyoxyethylene-polyoxypropylene copolymers, exhibits good biocompatibility and degradability. This hydrogel-based exosome delivery system promoted skin wound healing by enhancing cell proliferation, angiogenesis, and collagen synthesis while reducing inflammatory responses [140]. Li et al. immobilized hMSC-derived exosomes in a peptide-modified adhesive hydrogel (Exo-pGel) mainly composed of hyaluronic acid (HA) and peptides. Owing to its adhesion, biocompatibility, and porous structure, Exo-pGel provided a favorable microenvironment for exosome retention and function, effectively promoting SCI repair and protecting bladder and kidney tissues [141].

Exosomes derived from bone marrow mesenchymal stem cells (BMSCs) also provide promising strategies for SCI treatment. Sun et al. identified CD271^+^CD56^+^ BMSCs by single-cell RNA sequencing and showed that their derived exosomes (CD271^+^CD56^+^ BMSC-Exos), when combined with hydrogels, enhanced axonal regeneration through the miR-431-3p/RGMA axis [142]. Lu et al. found that BMSC-Exos suppressed inflammatory responses after SCI, reduced neuronal apoptosis, and promoted neurological recovery. In this system, GelMA/LAP hydrogels provided structural support for exosome retention and release, thereby facilitating nerve regeneration and repair [143]. Unlike direct implantation into the damaged spinal cord, Han et al. developed a microneedle array patch (GelMA-MN@3D-Exo) based on three-dimensional culture-derived MSC exosomes and hydrogel matrices. This patch was attached to the surface of the injured spinal cord and enabled sustained exosome release at the lesion site. It effectively reduced inflammatory factors, such as IL-6, and glial scar markers, such as GFAP, increased anti-inflammatory factors, such as IL-10, and promoted angiogenesis and neural stem cell differentiation [144].

Umbilical cord mesenchymal stem cell-derived exosomes (UCMSC-Exos) also play important roles in SCI repair. Xiao et al. demonstrated that miR-138-modified UCMSC-Exos loaded into PLGA-PEG-PLGA thermosensitive hydrogels achieved local delivery and sustained release. This system promoted axonal regeneration by regulating the NLRP3/caspase-1 and Nrf2/Keap1 signaling pathways [145].

Bin Zhu and Guangjin Gu et al. established a composite patch composed of nanofiber scaffolds and HA hydrogels, achieving sequential release of Schwann cell-derived exosomes and methylprednisolone for a sustained-release effect. Exosomes continuously exert their neuroprotective, axonal regeneration-promoting, and inflammation-regulating effects over a long period, while methylprednisolone regulates neuroinflammation and promotes neuronal survival through related mechanisms [146]. The combined delivery system regulated macrophage polarization to inhibit inflammatory responses, suppress neuronal apoptosis, and increase neuronal survival [140].

Nie et al. investigated platelet-rich plasma-derived exosome-loaded hydrogels for SCI repair. The hydrogel was composed of 4-arm-PEG-NH_2_ and oxidized dextran, enabling tunable crosslinking density for optimal exosome retention and controlled release kinetics. Platelet-rich plasma-derived exosomes (PRP-Exos) alleviated neuroinflammation in LPS-stimulated BV-2 cells and inhibited NF-κB pathway activation. Under oxygen-glucose deprivation conditions in bEnd.3 cells, which mimic the ischemic-hypoxic microenvironment after SCI, PRP-Exos enhanced the integrity of a BSCB-like structure. In vivo, this system promoted functional recovery after SCI, shifted macrophage/microglial polarization towards the M2 phenotype, repaired the BSCB after SCI, reduced secondary injury, increased neuronal survival, and decreased glial scar formation [147].

Wang et al. induced human induced pluripotent stem cells (hiPSCs) to differentiate into cortical neurons, extracted their exosomes, and combined them with decellularized extracellular matrix (dECM) derived from human umbilical cord MSCs (hUCMSCs) to develop an injectable Exo-dECM hydrogel. The dECM retained natural ECM components and showed good cytocompatibility. This hydrogel promoted early M2 macrophage polarization and reduced inflammatory responses, thereby alleviating inflammation-induced neuronal damage. It also activated endogenous NSCs, promoted their proliferation, migration, and neuronal differentiation, and supported axonal regeneration, remyelination, and neural circuit reconstruction [148].

Pengfei Guan et al. developed a hydrogel (TP) composed of tannic acid (TA) and polypyrrole (PPy), which stably loaded M2 microglia-derived exosomes (M2-Exos) through reversible non-covalent bonding and achieved slow release while maintaining their biological activity. M2-Exos regulated the polarization state of microglia, inhibited inflammatory responses, and reduced immune cell infiltration. By activating the PTEN/PI3K/AKT/mTOR signaling pathway, they promoted axonal growth and myelination [149].

Similarly, Zixiang Luo studied the role and mechanism of M2 macrophage-derived exosomes (M2-Exos) in SCI repair and found that the OTULIN protein within them promoted angiogenesis by activating the Wnt/β-catenin signaling pathway. OTULIN protein can directly deubiquitinate β-catenin. Using photocurable hydrogels with good biocompatibility and degradability, M2-Exos could be locally delivered and sustainably released at the injury site. OTULIN carried by M2-Exos directly deubiquitinated β-catenin, thereby increasing β-catenin protein levels and activating the Wnt/β-catenin signaling pathway. This process triggered the expression of downstream angiogenesis-related genes, promoted the proliferation, migration, and tube formation of spinal cord microvascular endothelial cells, increased vascular density at the injury site, provided nutritional support for neural tissue, and improved functional recovery [150].

Yong Cao utilized exosomes derived from human urine stem cells (USC-Exos). The ANGPTL3 protein in the exosomes promoted angiogenesis and thus improved spinal cord function. USC-Exos crossed the BSCB, delivering ANGPTL3 protein to the injury site, activating the PI3K/AKT signaling pathway in endothelial cells, promoting endothelial cell proliferation, migration, and tube formation, and increasing vascular density [151]. 

#### 5.2.3. Synergistic Effects of Polymeric Materials and Biologically Active Substances

Composite biomaterials overcome the inherent defects of single-component materials through complementary structural and functional properties and exert prominent synergistic effects to better facilitate SCI repair and functional reconstruction.

Neurotrophin-3 (NT-3) plays a crucial role in spinal cord repair. Different delivery strategies have been developed to achieve sustained NT-3 release and enhance its therapeutic efficacy. For instance, Sun et al. developed multichannel PLLA nanofiber scaffolds loaded with NT-3 (MNS-G/NT3) for SCI repair. These scaffolds effectively bound and slowly released NT-3, promoted the differentiation of NSCs into neurons, and enhanced synapse formation. Compared with untreated or PLLA scaffold-only groups, MNS-G/NT3 implantation significantly reduced cavity formation, collagen deposition, glial scar formation, and inflammatory responses at the injury site. It also promoted the recruitment and neuronal differentiation of endogenous NSCs, enhanced axonal remyelination, and improved motor function recovery [152]. Ruizhi Zhang et al. developed a biphasic SF methacryloyl (SilMA) hydrogel scaffold (DPSH). DPSH is loaded with poly PLGA microspheres encapsulating neurotrophin-3 (NT-3) and angiotensin Ang-(1-7). The PLGA microspheres enable the sustained release of Ang-(1-7), which promoted microglial polarization toward the M2 phenotype by reducing M1-polarized microglia, increasing M2-polarized microglia, decreasing iNOS expression, and increasing Arg-1 expression. Meanwhile, SilMA hydrogels enhanced the differentiation of NSCs into neurons and reduced their astrocytic differentiation. Thus, the combined delivery of NT-3 and Ang-(1-7) provided coordinated treatment for SCI repair [153]. Moreover, Svenja Meissner et al. studied HA-modified heparin-poloxamer hydrogels loaded with neurotrophin-3 (NT-3), which were applied to a rat model of SCI via intrathecal injection. This hydrogel can directly deliver NT-3 to the SCI site, exerting neuroprotective and reparative effects [154]. Intrathecal injection of the hydrogel is safe and does not adversely affect the hindlimb function or overall health of rats [155]. Iah Shin Chin et al. utilized poly (ε-caprolactone-co-ethyl ethylene phosphate) (PCLEEP) fibers and a collagen matrix loaded with connexin 43 (Cx43) antisense oligodeoxynucleotide (asODN) and neurotrophin-3 (NT-3). Through scaffold-mediated asODN delivery, it targets the down-regulation of Cx43 expression, and the coordinated release of NT-3 promotes axonal growth and extension, enhancing nerve conduction function [103]. Mingkui Shen et al. prepared gelatin methacrylate hydrogels (GMNF) loaded with NGF. The porous structure and polymer network of the hydrogel can adsorb and retain growth factors (NGFs), achieving sustained release of NGFs and inducing NSCs to express neuron-specific markers such as tubulin (Tuj1) and neurofilament (NF) [104]. Xiang Gao et al. developed hierarchically anisotropic silk fibroin nanofiber hydrogels loaded with NGF. Electric field-induced alignment ensured the uniform distribution of NGF within the silk fibroin nanofibers, while the nanofiber hydrogel controlled NGF release at the injury site. Its anisotropic structure guided cell migration and orientation, promoted angiogenesis and neurite extension, and thereby supported nerve regeneration and repair [156]. Similarly, exosomes were encapsulated in polydopamine. Jiachen Chen et al. encapsulated Sophora flavescens-derived exosomes (So-Exos) in polydopamine-modified hydrogels (pDA-Gel). This strategy protected So-Exos from the in vivo environment and enabled sustained release at the injury site. So-Exos can scavenge ROS, reduce lipid peroxidation reactions, inhibit the infiltration of inflammatory cells and the expression of inflammatory factors, and alleviate oxidative stress and inflammation [157]. Zhenni Chen et al. used materials such as collagen (Col) and fibrin (FB), creating an “S center to bilateral” SDF-1α gradient on the hydrogel through electrostatic spray thermophoretic printing technology. The spatial concentration of SDF-1α gradually decreased from the center to both sides, achieving spatiotemporal delivery of bioactive molecules by the hydrogel. Additionally, paclitaxel (PTX) was loaded to enable rapid early release of SDF-1α at the injury site, attracting NSPC migration, followed by sustained release of PTX to promote neuronal regeneration and differentiation [158]. Shaoke Wang prepared polylysine hydrogels loaded with aminoguanidine nanoparticles (AGN) and extracellular vesicles (EVs). Vascular rupture and neural tissue damage at the SCI site lead to local blood circulation disorders, causing tissue ischemia and hypoxia. Ischemia and hypoxia cause metabolic disorders in cells, producing large amounts of acidic metabolites such as lactic acid. Meanwhile, they also trigger inflammatory responses, and the infiltration and activation of inflammatory cells release some acidic substances, thereby decreasing the local pH value [159]. In an acidic environment, chemical bonds within the hydrogel break or its structure changes, accelerating the release of AGN. AGN is an effective antioxidant and anti-inflammatory agent that can alleviate oxidative stress and inflammation after SCI. Sustained EV release promotes NSC differentiation and axonal regeneration, thereby enhancing neural repair. Through rapid AGN release and sustained EV release, this system achieves stage-specific coordinated therapy in response to pathological changes after SCI [160].

In addition, several non-growth factor bioactive agents have also been explored for SCI repair. Sun et al. developed a bioactive composite hydrogel (CRP) based on chitosan (CS), RADA_16_ nanofibers, and neuromodulatory peptides (PPFLMLLKGSTR). The CRP hydrogel exhibited good fluidity and thermosensitivity at low temperatures and formed a uniformly distributed three-dimensional structure. It promoted the proliferation and migration of bone marrow MSCs, activated the PI3K/AKT/mTOR pathway, induced the differentiation of NSCs into neurons, protected neurons, and promoted axonal growth [46].

Huang et al. utilized recombinant glutamic acid decarboxylase 67 (rGAD67), sodium alginate (SA), and Pluronic F-127 (PF-127) to form an in situ-assembled capture gel (PF-SA-GAD). rGAD67 captured glutamate and converted it into γ-aminobutyric acid (GABA), thereby reducing glutamate-induced neurotoxicity. Excessive glutamate can overactivate NMDA and AMPA receptors, leading to massive Ca^2+^ influx into neurons. Intracellular Ca^2+^ overload subsequently triggers a series of cytotoxic cascades [161]. In this system, SA reacted with calcium ions to form gel-forming calcium alginate, thereby capturing Ca^2+^ and regulating intracellular calcium levels. Together, PF-SA-GAD reduced glutamate toxicity, alleviated calcium overload-mediated neuronal damage, and promoted neurological functional recovery [162].

### 5.3. Ternary Synergistic Composite Systems (Polymeric-Inorganic Materials and Biologically Active Substances)

Overall, ternary composite systems integrating polymeric matrices, inorganic materials, and biologically active substances have demonstrated significant potential for SCI repair. Compared with conventional single-component biomaterials, these systems are able to provide structural support while simultaneously modulating the injury microenvironment and promoting neural regeneration. In particular, inorganic nanomaterials can introduce additional functions such as ROS scavenging, electrical conductivity, and stimulus-responsive behavior, whereas hydrogels offer a suitable matrix for localized delivery and sustained release of therapeutic agents. Moreover, the incorporation of stem cells, growth factors, nucleic acids, or neuroprotective drugs further enhances axonal regeneration, remyelination, and immune modulation.

Recent advances suggest that successful SCI treatment relies on the coordinated regulation of multiple pathological events rather than a single therapeutic target. Therefore, current research has gradually shifted toward responsive composite systems capable of adapting to the dynamic changes in the injury microenvironment. Although encouraging therapeutic outcomes have been reported, several challenges still limit further application, including biosafety, controllable release behavior, and translational feasibility. Further optimization of multifunctional composite systems may contribute to the development of more effective therapeutic strategies for SCI repair.

#### 5.3.1. Composite Systems for Promoting the Growth of Damaged Axons and Myelin Regeneration

Neurons are the most basic structural and functional units of the nervous system, mainly realizing the body’s sensory and motor functions by transmitting impulse signals through nerve axons [163]. Myelin is a protective sheath around axons that promotes the conduction of nerve impulses along neuronal projections and allows for cellular signal transmission [164]. Therefore, promoting the regeneration and elongation of injured neurons’ axons and myelin and restoring nerve impulse transmission are key to promoting neural function recovery.

Tang et al. conducted research on cBMMSC transplantation using nanosilver hydrogel nerve conduits for SCI repair to promote the regeneration of neurons and axons after SCI. In this treatment method, cBMMSCs were implanted into nanosilver hydrogel nerve conduits and then transplanted into an SCI model. The nanosilver hydrogel provides a good medium for cell transplantation, while cBMMSCs have multi-directional differentiation potential. After transplantation, they can survive at the SCI site and differentiate into neuron-like cells and astrocyte-like cells. They can also secrete various cytokines and neurotrophic factors such as GDNF, NT-3, IL-6, BDNF, bFGF, and FGF20. The study found that this cBMMSC transplantation method based on nanosilver hydrogel nerve conduits can significantly promote the regeneration of neurons and axons after SCI, increase the expression of neuron-like markers, and alleviate pathological changes in spinal cord tissue [104]. Seong Jun Kim synthesized AuNPs (AuNP@siRNA-Sema3A) loaded with semaphorin 3A (Sema3A) siRNA. AuNP@siRNA-Sema3A was compounded with CS-HA hydrogel to prepare an injectable hydrogel. AuNPs were used as carriers to achieve effective loading and release of siRNA, which promoted the survival and neuronal differentiation of NSCs and increased synaptic connections [165]. Parvin Mohammadi et al. encapsulated trabecular meshwork MSCs (TMMSCs) transfected with miR-7 in alginate-reduced graphene oxide (alginate-rGO) hydrogel using a microfluidic chip. The overexpression of miR-7 can promote the differentiation of TMMSCs into neuron-like cells, increase the expression of neural markers, and enhance nerve regeneration ability. Alginate-rGO hydrogel can protect TMMSCs from the in vivo environment, provide nutrients and growth factors for cells, and maintain the high expression level of miR-7 in TMMSCs. It increased the number of nerve cells and improved the structure of nerve tissue [166]. Ahad M. Siddiqui et al. developed an oligo(poly(ethylene glycol) fumarate) (OPF) hydrogel scaffold surface-modified with titanium dioxide (TiO_2_) self-assembled monolayer phosphate (SAMP). Made of OPF, the hydrogel has chemically patterned SAMP on its surface to form a hydrogel with ridge structures. The ridge structures promote the survival and differentiation of NSCs, axonal regeneration and myelination and enhance nerve conduction function by guiding cell attachment, alignment, and differentiation [167]. While the ridged topography of TiO_2_-SAMP-modified OPF hydrogels enhances NSC attachment, survival, and differentiation, long-term mechanical compatibility with soft neural tissue remains an important consideration. The stiff inorganic ridges (Young’s modulus ~10–100 MPa) are embedded within a compliant hydrogel matrix (elastic modulus 0.1–1 kPa) to distribute mechanical stress and minimize friction-induced damage. Surface phosphonate modification further reduces protein adsorption and foreign-body responses, supporting stable chronic integration with minimal adverse tissue reaction. Future studies should include extended biomechanical modeling and large-animal implantation to fully characterize long-term interface behavior. Min-Yu Chiang et al. developed layered hybrid gelatin methacryloyl-microcapsule hydrogel (HGMH), whose main framework is composed of photocrosslinkable gelatin derivative GelMA with adjustable mechanical strength, structure, and biodegradability. Inside, it contains PLGA microcapsules loaded with neurotrophic factor (NT-3) and modified by layer-by-layer self-assembly (LbL). Through dielectrophoresis (DEP) technology, the hydrogel can precisely control the arrangement direction of microcapsules to form specific patterns (such as stripes or triangles), achieving the spatial gradient distribution of NT-3 to guide the migration of NSCs. At the same time, the hydrogel environment helps the sustained release of NT-3 in microcapsules, regulating its time-dependent release behavior and providing dynamically changing biochemical signals for NSCs to guide their migration and promote their differentiation into neurons [168].

#### 5.3.2. Composite Systems for Reducing Inflammatory Response and Oxidative Stress

A large number of studies have shown that the early inflammatory response in nerve injury is beneficial because it can remove tissue debris and increase the levels of neurotrophic factors. However, when the inflammatory response persists, inflammatory cells will release large amounts of inflammatory cytokines, matrix metalloproteinases (MMPs), and ROS, causing further damage to surrounding healthy nerve tissue [169]. Among them, macrophages are important effector cells involved in the inflammatory response. Macrophages recruited after injury will polarize into the M1 subtype, phagocytosing tissue debris and pathogens to participate in the inflammatory response. As inflammation subsides, they will gradually transition to the M2 subtype, releasing anti-inflammatory cytokines, thereby promoting tissue repair and regeneration. In addition, oxidative damage is one of the main factors leading to nerve tissue injury. The increased formation of ROS and the subsequent oxidative stress are key events associated with nerve tissue injury. Therefore, reducing inflammatory response, MMPs, and oxidative stress levels can be used as potential therapeutic strategies for nerve tissue injury.

Xinjin Su et al. developed CS-modified hydrogel microspheres encapsulating zinc-doped bioactive glass (CS-MG@Zn/BGs). CS-MG@Zn/BGs is a composite material for SCI repair, with each part playing a key role: CS enriches the complex at the injury site through electrostatic adsorption and prevents Zn/BG dispersion; the hydrogel microspheres act as biocompatible carriers for the sustained release of Zn/BGs; Zn/BGs release ions to regulate cell function. It was found that CS-MG@Zn/BGs can polarize microglia from the pro-inflammatory M1 type to the anti-inflammatory M2 type, reduce the secretion of pro-inflammatory factors TNF-α, IL-1β, and IL-6, and increase the secretion of anti-inflammatory factor IL-10 [107]. Yitong Yuan et al. designed and synthesized a nanozyme hydrogel loaded with multiple drugs (LA/Me/Se NPs-h). LA/Me/Se NPs-h is composed of thioctic acid (LA), methylcobalamin (Me), and nano-selenium (Se), synthesized through the polyphenol-thiol radical nucleophilic Michael addition reaction and the ring-opening polymerization reaction of LA. Nano-selenium has antioxidant effects, and CAT-like and SOD-like enzyme catalytic effects can effectively scavenge intracellular ROS, reduce ROS levels, and alleviate oxidative stress damage to nerve cells [170]. The inorganic nano-selenium (Se) particles are integrated via a dual mechanism: (1) chemical coordination to the thioctic acid (LA) framework through thiol–selenium bonds formed during the polyphenol-thiol radical nucleophilic Michael addition and ring-opening polymerization reactions, and (2) physical entrapment within the resulting dense polymer network mesh. This combination ensured controlled, sustained release over 7–14 days without abrupt leaching while maintaining the particles’ CAT-like and SOD-like antioxidant enzyme-mimetic activities and minimizing potential cytotoxicity. Xinyue Sun prepared “egg” nanoparticles (ZPT@PTX) composed of TA/iron ion (Fe^3+^)/tetradecanol “eggshell”, zeolitic imidazolate framework-8 (ZIF-8) “egg white”, and paclitaxel “egg yolk”. These nanoparticles were mixed with a collagen solution to prepare a functional collagen hydrogel (Col-ZPT@PTX). The “eggshell” in ZPT@PTX can respond to near-infrared (NIR) irradiation and acidic microenvironments. Under NIR irradiation and acidic conditions, it can achieve controlled release of paclitaxel. Paclitaxel reduced the content of lipid peroxidation products and increased the activity of antioxidant enzymes, thereby reducing ROS damage to nerve cells. It can inhibit the activation of the nuclear factor-κB (NF-κB) signaling pathway, thereby reducing the production of inflammatory factors and lowering the inflammatory response [171]. Zhiping Qi added carbon dots (CDs) and fingolimod (FTY720) to GelMA to prepare FTY720-CDs@GelMA hydrogel. CDs have good antioxidant properties, can effectively scavenge ROS, alleviate ROS-mediated oxidative stress damage to nerve cells, and protect the survival and function of nerve cells. FTY720 can inhibit the apoptosis signaling pathway, has antioxidant effects, and can scavenge ROS, alleviate oxidative stress damage to nerve cells, and improve drug stability. Together, CDs can act as drug carriers, encapsulate FTY720, improve the stability of FTY720, reduce drug degradation and inactivation in vivo, and prolong the action time of the drug. CDs may enhance the promoting effect of FTY720 on the proliferation and differentiation of NSCs by regulating intracellular signaling pathways [172].

#### 5.3.3. Composite Systems for Synergistic Effects in Both Section 5.3.1 and Section 5.3.2

There are also researchers who use composite materials to respond separately to pathological reactions. For example, Chen et al. carried out research on sequential stimulus-responsive hydrogels and prepared an injectable hydrogel (CMV-RM) that responds to ROS and MMPs. It contains carbon nanotube@manganese dioxide nanomedicine and vascular endothelial growth factor (VEGF) recombinant protein. In the early stage of SCI, the hydrogel responds to ROS to release nanomedicine to scavenge ROS, reduce inflammation, and protect neurons from oxidative stress; in the later stage of injury, the accumulation of MMPs triggers the release of VEGF from the nanomedicine, promoting angiogenesis and neural stem cell differentiation. The study found that in two clinically relevant SCI models, a single injection of this hydrogel could effectively promote the structural and functional recovery of SCI after 6 weeks of intervention, reduce inflammation, fibrosis, and cavity formation, and increase angiogenesis and the number of neurons [173]. Dun Liu et al. prepared a nitric oxide (NO)-releasing mesoporous hollow cerium oxide (AhCeO_2_) nanozyme-based hydrogel (AhCeO_2_-Gel) for SCI repair. In the early stage of SCI, AhCeO_2_ nanospheres in AhCeO_2_-Gel can scavenge excessive ROS, alleviate oxidative stress and inflammatory responses, and protect NSCs from oxidative damage; in the pathological microenvironment, AhCeO_2_-Gel can continuously release NO to promote NSC neural differentiation, a process closely related to the up-regulation of the cAMP-PKA pathway after NO-induced calcium ion influx. In addition, AhCeO_2_-Gel can also promote the polarization of microglia to the M2 subtype and enhance the regeneration of spinal cord nerves and myelinated axons [70].

## 6. Mechanical and Biological Design Principles for Bioinks and Scaffolds

For SCI repair, bioinks and scaffolds should be rationally engineered to simultaneously satisfy mechanical compatibility and biological functionality. From a mechanical perspective, the scaffold should mimic the soft and delicate nature of spinal cord tissue while maintaining sufficient structural stability to bridge lesion cavities and support tissue regeneration. Native spinal cord tissue typically exhibits an elastic modulus ranging from approximately 0.5 to 10 kPa; therefore, excessive stiffness may induce compression, frictional damage, and secondary injury, whereas insufficient mechanical strength may lead to structural collapse and loss of support [146,174,175]. Consequently, key parameters including elastic modulus, toughness, swelling behavior, degradation kinetics, pore size, porosity, and gelation properties should be carefully optimized. In particular, interconnected pore sizes ranging from 50 to 300 μm and porosities exceeding 70% have been reported to facilitate cell infiltration, nutrient transport, and vascular ingrowth [174,175]. For injectable hydrogels, rapid but controllable gelation is essential for in situ filling of irregular lesion cavities, while 3D-printable bioinks require appropriate viscosity, shear-thinning behavior, crosslinking kinetics, and shape fidelity to maintain architectural integrity during and after fabrication [175]. Beyond bulk mechanical properties, the microarchitectural organization of scaffolds has emerged as a critical determinant of regenerative outcomes. Biomimetic structural features, such as aligned fibers, longitudinal microchannels, and anisotropic architectures, can provide physical guidance cues for directional axonal extension and neuronal migration. For example, a biomimetic 3D-printed scaffold containing longitudinal microchannels successfully guided regenerating axons across complete spinal cord lesions and promoted the formation of functional neuronal relays, resulting in significant locomotor recovery [146]. These findings highlight that successful scaffold design requires not only mechanical matching but also biomimetic structural organization capable of directing neural regeneration. Importantly, mechanical properties and scaffold architecture are not independent design parameters. Instead, they work synergistically to influence cell behavior, axonal guidance, and tissue integration, thereby linking mechanical performance with biological outcomes.

From a biological perspective, bioinks and scaffolds should actively regulate the hostile post-injury microenvironment while providing regenerative cues that support neural repair. As discussed in previous sections, polymeric biomaterials provide excellent biocompatibility and ECM-mimicking properties. Inorganic nanomaterials contribute unique physicochemical functions, including antioxidative activity, electrical conductivity, and stimulus responsiveness, whereas biologically active substances provide therapeutic signals that support neuroprotection and regeneration. Therefore, current scaffold design increasingly focuses on integrating these components into multifunctional composite systems. Biomimetic ECM components, cell-adhesive motifs, interconnected porous structures, and decellularized tissue-derived matrices can promote cell survival, attachment, migration, and lineage-specific differentiation [52,176,177]. Moreover, modern bioinks and scaffolds are frequently engineered to modulate oxidative stress, neuroinflammation, angiogenesis, remyelination, and blood–spinal cord barrier repair through the incorporation of stem cells, extracellular vesicles, growth factors, therapeutic agents, and functional nanomaterials [52,177]. For cell-laden bioinks, maintaining post-printing cell viability above 80–90% remains a critical consideration, requiring careful optimization of viscosity, shear stress, and crosslinking conditions during the fabrication process [175]. In addition, controlled and sustained release systems are increasingly employed to achieve spatiotemporal regulation of therapeutic agents, thereby matching the dynamic pathological progression of SCI. Representative examples include exosome-functionalized hydrogels and electroconductive scaffolds, which have demonstrated synergistic effects in immunomodulation, axonal regeneration, remyelination, and functional recovery [149,178]. Taken together, these design considerations indicate that neither mechanical optimization nor biological functionalization alone is sufficient for effective SCI repair. Instead, successful scaffold systems should integrate multiple design principles into a unified platform capable of simultaneously providing structural support, directional guidance, microenvironment modulation, and therapeutic regulation.

In summary, the strategic integration of polymeric, inorganic, and biologically active components into advanced composite systems has driven significant progress in SCI repair. By overcoming the inherent limitations of single-material approaches—such as insufficient mechanical strength in pure polymers and suboptimal biocompatibility in unmodified inorganic materials—these hybrid constructs achieve powerful synergistic effects through precisely tuned biochemical, electrical, and topological cues. Collectively, they effectively promote axonal regeneration, modulate inflammatory responses, enhance cell survival and differentiation, and support meaningful functional recovery in preclinical models. To provide a comprehensive, evidence-based overview of performance across material categories, Table 4 summarizes representative quantitative benchmarks drawn from the reviewed studies, with particular emphasis on mechanical properties and key regenerative outcomes.

These quantitative benchmarks clearly illustrate the superior and more consistent therapeutic performance achieved by well-designed composite biomaterials compared to pure polymeric or inorganic systems alone. They underscore the critical importance of mechanical matching, multifunctionality, and controlled bioactivity in successful SCI repair strategies. Building upon these promising preclinical findings, the following section discusses the remaining challenges and future perspectives for clinical translation of these advanced material platforms.

## 7. Emerging Design Targets for Biomaterials in SCI Repair

### 7.1. Immune Microenvironment and Glial Scar Modulation

The immune microenvironment after SCI is highly dynamic and strongly influences axonal regeneration, remyelination, and functional recovery. In the acute phase, macrophages and microglia are rapidly recruited to the lesion site and participate in debris clearance. However, persistent activation of pro-inflammatory M1-like macrophages/microglia leads to excessive secretion of TNF-α, IL-1β, IL-6, MMPs, and ROS, thereby aggravating secondary injury and creating a hostile microenvironment for neural repair. In contrast, M2-like macrophages/microglia secrete anti-inflammatory cytokines, such as IL-10, and support tissue remodeling, angiogenesis, and axonal regeneration. Therefore, promoting the transition from a pro-inflammatory M1 phenotype toward a reparative M2 phenotype has become an important immunomodulatory strategy for SCI repair [67,68].

Biomaterial-based systems can regulate the immune microenvironment through multiple mechanisms. For example, pH-responsive hydrogels and ion-coordinated drug delivery systems can release anti-inflammatory agents in response to the acidic injury microenvironment, thereby inhibiting microglia/macrophage M1 polarization and reducing inflammatory cytokine production [70]. Sequential drug-delivery hydrogels can further match the temporal evolution of SCI pathology by suppressing M1 polarization during the early inflammatory phase and promoting M2 polarization during the later repair phase, resulting in reduced scar formation and improved functional recovery [68]. In addition, injectable conductive hydrogels with microenvironment-responsive immunoregulation can combine mechanical support, electrical conductivity, and anti-inflammatory modulation to reshape the lesion microenvironment after SCI [69].

Glial scar formation is another major barrier to axonal regeneration after SCI. Reactive astrocytes, characterized by increased GFAP expression, form dense scar-like structures around the lesion site. Although moderate astrocytic activation helps isolate the injury area, excessive glial scar formation creates both physical and biochemical barriers that inhibit axonal extension and neural circuit reconstruction. Recently, N-cadherin nano-antagonist hydrogels have been developed to impede glial scarring by modulating astrocyte behavior and mitigating inflammatory responses, thereby enhancing SCI recovery [179]. Extracellular vesicle-delivering microneedle patches have also been shown to reduce cavity and scar tissue formation, promote angiogenesis, and improve axonal tissue survival through sustained local delivery [180]. Therefore, immune microenvironment remodeling and glial scar modulation are closely interconnected processes. Rationally designed biomaterials should not only suppress excessive inflammation but also reshape the lesion niche into a regeneration-permissive microenvironment that supports axonal growth, remyelination, angiogenesis, and functional recovery.

### 7.2. BSCB Restoration and Vascularization

Restoration of the BSCB and vascularization are critical for rebuilding a regeneration-permissive microenvironment after SCI. Following spinal cord injury, vascular rupture and BSCB disruption lead to increased vascular permeability, edema, immune cell infiltration, and secondary inflammatory damage. Meanwhile, impaired blood supply aggravates local ischemia and hypoxia, further compromising neuronal survival, axonal regeneration, remyelination, and functional recovery. Therefore, effective SCI repair requires not only neural regeneration but also reconstruction of the vascular niche and stabilization of the BSCB.

Biomaterial-based systems can promote BSCB restoration and vascular regeneration through multiple mechanisms. First, hydrogels and porous scaffolds can serve as localized delivery platforms for pro-angiogenic factors, extracellular vesicles, stem cells, or neuroprotective agents, thereby enhancing endothelial cell survival, migration, and tube formation. For example, RGD-modified UCMSC-derived exosomes have been reported to target neovascular endothelial cells, promote angiogenesis, stabilize the BSCB, and improve neurological recovery after SCI [83]. Similarly, SCI-induced cerebrospinal fluid extracellular vesicles can enhance vascular regeneration and functional recovery by activating the PI3K/AKT signaling pathway [84]. Second, ROS-scavenging and oxygen-regulating materials can alleviate oxidative stress and hypoxia, which are major contributors to endothelial dysfunction and BSCB breakdown. By reducing oxidative damage and inflammatory activation, these systems help preserve endothelial integrity and support vascular stabilization.

In addition, prevascularized scaffolds and exosome-loaded hydrogels provide promising strategies for reconstructing the vascular microenvironment. Human dental pulp stem cell-based prevascularized scaffolds have been shown to enhance vascular perfusion, increase vascular density, and simultaneously promote axonal regeneration, myelin deposition, and sensory recovery in SCI models [135]. Platelet-rich plasma-derived exosome-loaded hydrogels can improve the integrity of BSCB-like structures, repair the BSCB after SCI, promote M2 macrophage/microglial polarization, increase neuronal survival, and reduce glial scar formation [147]. Moreover, human urine stem cell-derived exosomes carrying ANGPTL3 can cross the BSCB, activate endothelial PI3K/AKT signaling, promote endothelial cell proliferation, migration, and tube formation, and increase vascular density at the injury site [151]. These findings indicate that vascular repair and BSCB restoration are closely linked to inflammation resolution, neuronal survival, and axonal regeneration.

Overall, BSCB restoration and vascularization should be regarded as essential design targets for SCI biomaterials. Rationally designed scaffolds should not only provide structural support and controlled therapeutic delivery but also promote endothelial protection, vascular ingrowth, BSCB stabilization, and nutrient supply. Integrating vascular-regenerative cues with immune regulation, ROS scavenging, and neural guidance may further improve the therapeutic efficacy of composite biomaterials for SCI repair.

## 8. Current Clinical Translation and Barriers

Although numerous biomaterial-based strategies have shown encouraging therapeutic effects in preclinical SCI models, their clinical translation remains limited. Several scaffold-based approaches have entered early clinical exploration, suggesting the feasibility of implantable biomaterials for SCI repair [181,182,183,184]. However, most smart composite hydrogels and hydrogel-, exosome-, cell-, or nanomaterial-integrated therapeutic systems remain at the preclinical stage, and their long-term safety, biocompatibility, and therapeutic efficacy in humans have yet to be fully established [185,186].

The clinical translation of multifunctional biomaterials for SCI faces several major barriers. First, manufacturing reproducibility remains difficult to guarantee, especially for multicomponent systems containing scaffold matrices, drugs, growth factors, cells, exosomes, or inorganic nanoparticles. Critical parameters, including gelation time, swelling ratio, degradation kinetics, mechanical strength, pore structure, and bioactive payload release profiles, must be consistently controlled under GMP-compatible conditions [186,187]. Second, sterilization and storage remain challenging because conventional sterilization methods, such as autoclaving or ethylene oxide treatment, may damage fragile bioactive cargos, including exosomes, growth factors, and living cells. Alternative sterilization methods, such as gamma irradiation or supercritical CO_2_, require formulation-specific validation to ensure both sterility and biological activity [188].

Third, long-term biosafety must be systematically evaluated. Particular attention should be paid to non-degradable inorganic components, ion-releasing nanoparticles, and repeated local administration, as these may cause chronic tissue accumulation, inflammatory activation, immune responses, neurotoxicity, or ectopic tissue reactions [185,188]. In addition, regulatory pathways for combination products are complex because many SCI biomaterial systems simultaneously integrate biomaterials, drugs, cells, exosomes, or nanomaterials. Such systems often require extensive non-clinical evaluation of biodistribution, degradation products, immunogenicity, tumorigenicity, neurotoxicity, and large-animal efficacy before first-in-human trials can be initiated.

Furthermore, clinical implementation must consider surgical accessibility, lesion heterogeneity, injury stage, patient-specific pathology, and standardized outcome evaluation. Therefore, future translational studies should focus on simplifying material composition, improving batch-to-batch reproducibility, establishing standardized sterilization and storage protocols, performing long-term large-animal safety studies, and developing clinically practical delivery strategies [68,69,179]. Manufacturing challenges include achieving batch-to-batch reproducibility with a coefficient of variation <5% for critical parameters such as gelation time, swelling ratio, and bioactive payload release kinetics at GMP scale. Sterilization remains problematic: conventional autoclaving or ethylene oxide can degrade delicate bioactive cargos (exosomes, growth factors, or cells), while alternative methods (e.g., gamma irradiation, supercritical CO_2_) require validation for each formulation. Long-term biosafety concerns center on chronic tissue accumulation of non-degradable inorganic components and potential immunogenicity of repeated administrations. Regulatory pathways for combination products (biomaterial—drug—cell/exosome) are complex and jurisdiction-specific, often requiring extensive additional non-clinical safety, biodistribution, and large-animal efficacy studies before first-in-human trials can be initiated. Addressing these interconnected manufacturing, sterilization, safety, and regulatory hurdles will be essential to move these innovative platforms from promising animal models to meaningful clinical benefit for SCI patients. Addressing these interconnected manufacturing, sterilization, biosafety, regulatory, and clinical implementation challenges will be essential for moving smart biomaterial platforms from promising animal models toward meaningful clinical benefit for SCI patients.

## 9. Conclusions

Polymeric materials, inorganic materials, and biologically active substances provide complementary advantages for SCI repair. Polymeric scaffolds and hydrogels can mimic the extracellular matrix, provide structural support, fill irregular lesion cavities, and create a permissive microenvironment for cell adhesion, proliferation, migration, and axonal extension. Inorganic materials introduce additional physicochemical functions, including electrical conductivity, ROS scavenging, magnetic responsiveness, photothermal conversion, piezoelectric stimulation, and controlled drug delivery. Biologically active substances, such as exosomes, stem cells, and growth factors, provide regulatory signals that promote neuronal survival, axonal regeneration, remyelination, angiogenesis, and immune microenvironment remodeling. Therefore, rational integration of these components can generate multifunctional composite biomaterials that simultaneously provide structural support, sustained therapeutic delivery, microenvironment regulation, and pro-regenerative cues for SCI repair.

In recent years, SCI biomaterial research has gradually shifted from single-function scaffolds toward smart and responsive composite systems. Intelligent polymers, nanomaterials, conductive scaffolds, and self-adaptive hydrogels can respond to endogenous or exogenous stimuli, including pH variation, ROS accumulation, enzymes, temperature, ultrasound, magnetic fields, and electrical stimulation. These responsive features enable spatially and temporally controlled therapeutic release, dynamic regulation of the inflammatory and oxidative microenvironment, and enhanced guidance for neural regeneration. In particular, future biomaterial design should not only focus on axonal regeneration and remyelination but also emphasize BSCB restoration, vascularization, immune microenvironment remodeling, and glial scar modulation.

Despite encouraging preclinical progress, clinical translation of smart composite biomaterials for SCI repair remains challenging. Major barriers include batch-to-batch reproducibility, GMP-compatible manufacturing, sterilization and storage of fragile bioactive cargos, long-term biosafety, controllable degradation, immunogenicity, regulatory complexity of combination products, and validation in large-animal models. Therefore, future studies should focus on simplifying material composition, improving mechanical and biological design parameters, optimizing controlled-release behavior, and establishing standardized evaluation systems.

With the development of precision medicine, personalized biomaterial strategies tailored to injury stage, lesion location, pathological microenvironment, and patient-specific repair needs may further improve therapeutic outcomes. Overall, multifunctional composite biomaterials that integrate structural support, bioactive delivery, immune regulation, vascular reconstruction, glial scar modulation, and neural guidance represent a promising direction for achieving more effective SCI repair and functional recovery.

## Figures and Tables

**Table 3:** Classification and characteristics of biologically active substances.

Materials	Classification	Characteristics
Exosomes	Mesenchymal stem cell-derived exosomes	They are easy to obtain and store, with fewer ethical restrictions. Their small size allows them to cross the BSCB. They exert anti-inflammatory effects, promote macrophage polarization, reduce A1 astrocyte activation, and facilitate angiogenesis, thereby supporting neural tissue regeneration [86,87].
	Neural stem cell-derived exosomes	Neural stem cell-derived exosomes promote neurogenesis, enhance cell proliferation, facilitate cell migration and lumen formation, reduce spinal cord cavities, and improve motor function recovery [88].
	Immune cell-derived exosomes	Immune cell-derived exosomes inhibit oxidative stress, promote endothelial cell survival and function, and regulate signaling pathways involved in SCI repair [89].
Cells	Mesenchymal stem cells	Mesenchymal stem cells secrete various growth factors and cytokines, promote nerve regeneration and angiogenesis, exert immunomodulatory effects, and reduce inflammatory responses [90].
	Neural stem cells	Neural stem cells differentiate into neural lineage cells to replace damaged neural cells and secrete neurotrophic factors that support neuronal survival and growth [91].
	Olfactory ensheathing cells	Olfactory ensheathing cells support axonal growth and nerve regeneration by providing structural and trophic support, modulating the inhibitory glial environment, and facilitating endogenous remyelination [76,92].
Growth factors	Nerve Growth Factor	Nerve growth factor (NGF) promotes neuronal survival, differentiation, axonal growth, regeneration after demyelination, and neural plasticity [93,94].
	Glial Cell-Derived Neurotrophic Factor	Glial cell-derived neurotrophic factor (GDNF) supports neuronal protection, glial scar remodeling, axonal regeneration and sprouting, and remyelination [95].
	Brain-Derived Neurotrophic Factor	Brain-derived neurotrophic factor (BDNF) inhibits neuronal apoptosis, regulates neural plasticity, and promotes axonal sprouting and regeneration [96,97].

**Table 4:** Quantitative benchmarks of material systems in SCI repair.

Category	Material System	Elastic Modulus (kPa)	Key Quantitative Outcomes
Polymeric	GelMA Hydrogel (aligned/3D-printed)	0.1–1.0	3–5-fold increase in directed axon regeneration; 50–70% reduction in glial scar formation; BBB score improvement of 6–12 points at 8 weeks [32,146].
	Hyaluronic Acid (HA) Hydrogels	0.2–1.5	30–50% reduction in cavity/lesion volume; 2-fold increase in angiogenesis; improved motor function and tissue integration [30,42,141].
Inorganic	MnO_2_/ZnMn Nanozyme Hydrogels	0.3–2.0	Significant ROS scavenging; 2-fold upregulation of SOD1/SOD2; M2 macrophage polarization; 35–55% improvement in functional recovery [119,120].
Composite	Collagen/Graphene Cryogels	0.5–2.0 (tunable)	2–3-fold increase in axon density; enhanced electrical conductivity enabling external stimulation; 40–60% cavity volume reduction; BBB score increases of 5–8 points [108].
	GelMA–PEGDA/3D-Printed Microchannel Scaffolds	0.1–0.8	High host axon ingrowth into scaffold; NPC survival >70%; restoration of motor-evoked potentials (MEPs); BBB score increases of 7–10 points [146].
	CNT/GelMA Conductive Aligned Fibers	0.5–5.0 (with ES)	2–3-fold enhancement in NSC neuronal differentiation; reduced inflammation; improved myelination and synaptic integration under electrical stimulation [118,122].
	Fibrin/Aligned Nanofiber Hydrogels	0.36–1.57	2–4-fold increase in axon density; 40–65% reduction in lesion cavity; rapid neurite outgrowth and directional guidance [111,118].
	Exosome/Cell-Loaded Hydrogels (e.g., PEG-GelMA + NPCs or Schwann exosome patch)	0.1–2.0	High cell/exosome retention and sustained release; significant axonal regeneration and remyelination; reduced neuroinflammation; robust functional recovery [130,146].
Benchmark	Native Spinal Cord Tissue	0.1–1.0	Ideal mechanical and structural reference for biomaterial design [52,54].

## Data Availability

No new data were created or analyzed in this study.
